# [Retracted] Matrine-induced apoptosis in Hep3B cells via the inhibition of MDM2

**DOI:** 10.3892/mmr.2026.13963

**Published:** 2026-07-13

**Authors:** Ning Zhou, Jiequn Li, Ting Li, Guangshun Chen, Zhongqiang Zhang, Zhongzhou Si

Mol Med Rep 15: 442–450, 2017; DOI: 10.3892/mmr.2016.5999

Following the publication of the above paper, the following areas of concern associated with the western blot data featured in Figs. 3, 4 and 5 were drawn to the Editor's attention by a concerned reader, essentially involving the apparent incorrect assembly of blots within these figures and duplications of data: i) Control blots were shared between Figs. 3A and 5A, where the experimental conditions were reported to be different; ii) MDM2 blots may have been featured incorrectly in Figs. 4B and 5A, as the control blots/reported experimental conditions featured in these figure parts appeared to be the same; and iii) there was also the probable duplication of protein bands of interest comparing Fig. 5A and B. A subsequent analysis of the data in this paper performed by the Editorial Office also revealed the potential re-use of data in a paper that was published a year later, also in the journal *Molecular Medicine Reports*, albeit no authors were held in common comparing these papers, and that paper has since been retracted on account of the re-use of data with another article:

Given the identification of the above problems associated with the assembly of data in three of the figures in this paper, the Editor has decided that it should be retracted from the Journal on account of a lack of confidence in the data. The authors were asked for an explanation to account for these concerns, but the Editorial Office did not receive a reply. The Editor apologizes to the readership for any inconvenience caused.

